# Diagnosis of an Inguinal Hernia after a Blunt Inguinal Trauma with an Intestinal Perforation

**DOI:** 10.1155/2014/653847

**Published:** 2014-03-09

**Authors:** Farès Moustafa, Julien Avouac, Marie-Aude Vaz, Jeannot Schmidt

**Affiliations:** ^1^CHU Clermont-Ferrand, Pôle SAMU-SMUR-Urgences, Hôpital Gabriel Montpied, 58 rue Montalembert, BP 69, 63003 Clermont-Ferrand, France; ^2^CHU Clermont-Ferrand, Service de Chirurgie Vasculaire, Hôpital Gabriel Montpied, 63003 Clermont-Ferrand, France; ^3^CHU Clermont-Ferrand, Service de Radiologie A, Hôpital Gabriel Montpied, 63003 Clermont-Ferrand, France

## Abstract

*Introduction*. Inguinal hernias are very common in men. A clinical exam can do the diagnosis easily. But bowel perforation inside an inguinal hernia caused by a directly blunt trauma is rare and can have important consequences. Up to now, there have been a few case reports that described blunt injury to the inguinal area causing traumatic perforation of the bowel in the inguinal hernia. *Case Report*. We present a case of a 45-year-old Eastern European man with a small perforation of ileal bowels and a peritonitis after direct blunt trauma to the inguinal hernia region, with no inguinal hernia known by the patient, and show how the diagnosis can be difficult. *Conclusion*. This case shows that external forces, that may seem too trivial to cause intraperitoneal injury, can cause significant injury when applied to a patient with a hernia and shows how a careful examination, with the help of an abdominal CT scan, is important even if the patient do not seem to have an inguinal hernia.

## 1. Introduction

Inguinal hernias are very common in men. A clinical exam can do the diagnosis easily. Treatment is often a surgical intervention because of the risk of intestinal incarceration and strangulation.

But bowel perforation inside an inguinal hernia caused by a directly blunt trauma is rare and can have important consequences.

Up to now, there have been a few case reports that described blunt injury to the inguinal area causing traumatic perforation of the bowel in the inguinal hernia [1–5].

This case report emphasizes the difficult diagnosis and the potential clinical complication of an unknown inguinal hernia if a blunt trauma occurs in the inguinal area.

## 2. Case Presentation

During a handball match, a 45-year-old Eastern European man with a medical history of appendicectomy and no inguinal hernia known suffered trauma from another player's knee onto the right inguinal area.

After this trauma, he presented a collapse for few seconds and developed intense pain in the hypogastrium. He was immediately taken by the emergency medical service and transferred to the Emergency Department, where he has the following vital signs: heart rate 90 beats/min, blood pressure 104/61 mmHg, and temperature 37°C.

Abdominal examination revealed diffuse pain, absence of intestinal sounds, and involuntary muscular resistance but no inguinal hernia.

Laboratory tests showed a hematocrit of 46.5%, leukocytes 6730/mm^ 3^, serum creatinine 89 *μ*mol/L, and blood urea nitrogen 8.5 mmol/L.

**Figure 1:**
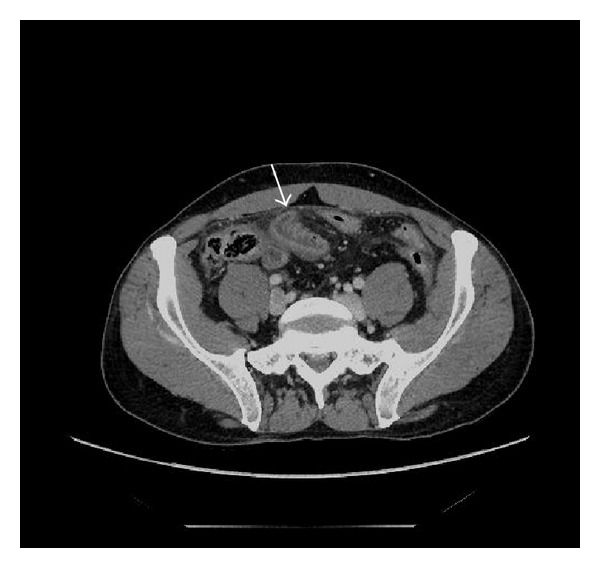
Contrast enhanced axial CT of the abdomen in a 45-year-old male with an inguinal trauma. Bowel wall thickening and enhancement.

**Figure 2:**
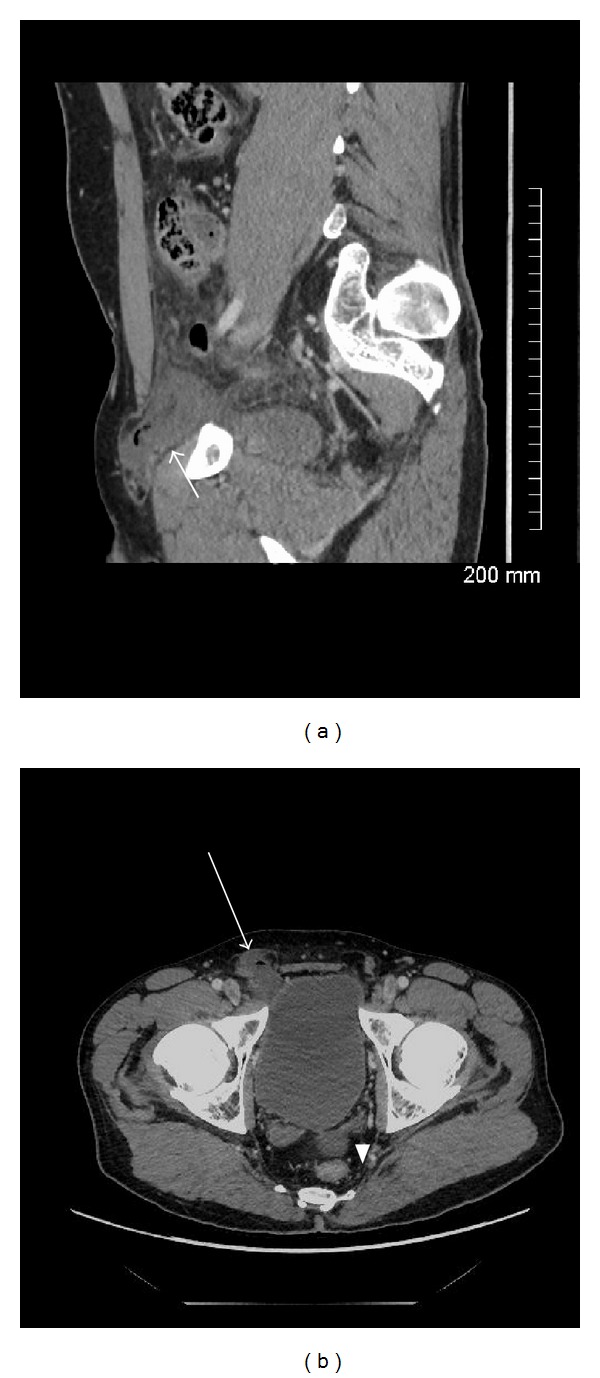
Contrast enhanced coronal (a) and axial (b) CT of the abdomen in a 45-year-old male with an inguinal trauma, showing right inguinal hernia, with extraluminal air (white arrow) and fluid in the peritoneal cavity (arrowhead).

**Figure 3:**
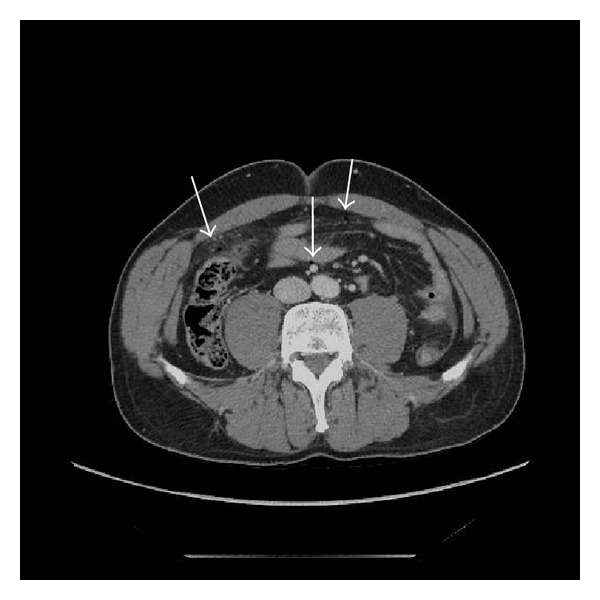
Contrast enhanced axial CT of the abdomen in a 45-year-old male with an inguinal trauma, showing free intraperitoneal air bubbles.

Ultrasounds showed no fluid in the peritoneal cavity. So an abdominal CT scan with intravenous contrast was performed and showed a right inguinal hernia, a pneumoperitoneum in the abdomen and in the right inguinal area, with a good bowel wall enhancement (Figures 1, 2, and 3).

Laparotomy was performed in emergency, revealing spontaneous reduction of the inguinal hernia with a small perforation of ileal bowels and peritonitis. A peritoneal cavity lavage was performed and resection of the intestinal lesion was done.

Throughout surgery, the patient went better with decreasing septic syndrome, normal intestinal sounds, and food reintroduction. The abdominal drain was taken off 3 days after the surgery and the silicon blade 9 days after.

The patient could be discharged from the hospital at 10 days. The right inguinal hernia was repaired three months later.

## 3. Discussion

In our case, perforation of the ileum occurred as a consequence of a blunt trauma on the inguinal area to a patient with an unknown inguinal hernia.

Twenty-five percent of men and 2% of women develop inguinal hernias in their lifetime [6]. Incarceration and strangulation of the intestinal structures are the most frequent reported complications of unrepaired inguinal hernia. Overall, intestinal and mesenteric injury represent 5% of patients with blunt abdominal trauma [7].

If intestinal perforation in patients with preexisting hernias has been reported from blunt trauma to the abdomen, blunt trauma directly to the inguinal hernia more rare [3, 8, 9].

In men older than 45 years with hernias, blunt abdominal trauma with intestinal perforation has been reported to occur more commonly [4]. Also patient with right inguinal hernias and also with femoral, perineal, and incisional hernias have more frequently intestinal perforation [3].

The deceleration and compression forces induce perforation [7]. Stretching and linear tearing between fixed and movable objects occur in the deceleration injuries. For the compression forces, increasing of the intraluminal pressure causes rupture.

Intraluminal pressure can be increased by the increase of intra-abdominal pressure, and intestinal loops overlying the hernia aperture can blow out over the aperture [3]. Intestinal loops trapped inside a hernia are also susceptible to perforation. The adult inguinal canal is 4 cm long and is bounded anteriorly by the external oblique aponeurosis muscle, posteriorly by the transversalis fascia and the aponeurosis of the transversus abdominis muscle, superiorly by the internal abdominal oblique and transversus abdominis muscles, and inferiorly by the inguinal and lacunar ligaments.

In 1995, Reynolds [1] explained how the incoming and outgoing loops bowel are compressed when a direct trauma to an inguinal hernia occurs; then additional pressure applied to the sealed loop generated enough intraluminal pressure to cause a perforation. This direct trauma to an inguinal hernia can give spikes of pressure greater than 300 mm Hg, when only 150–260 mmHg is enough to induce an intestinal loops rupture.

In our case report the fact that the patient did not know that he had an inguinal hernia made the diagnosis difficult. So the evaluation of an abdominal pain after a blunt trauma needs a systematic approach with a good clinical evaluation by the same physician and some medical imaging to help the physician.

Even if simple X-rays can show free intraperitoneal air, abdominal contrast enhanced CT ought to be the choice of the physician.

In fact, on CT, bowel perforation is suspected because of the presence of pneumoperitoneum or free fluid but we must keep in mind that the chances of detecting these signs increase as time elapses [10].

## 4. Conclusion

This case shows that external forces, that may seem too trivial to cause intraperitoneal injury, can cause significant injury when applied to a patient with a hernia and shows how a careful examination, with the help of an abdominal CT scan, is important even if the patient does not seem to have an inguinal hernia.
